# Supplementary material for: The Influence of Hop Latent Viroid (HLVd) Infection on Gene Expression and Secondary Metabolite Contents in Hop (Humulus lupulus L.) Glandular Trichomes
Source: Plants (Basel). 2021 Oct 26;10(11):2297. doi: 10.3390/plants10112297 (PMC8617911; doi:10.3390/plants10112297)
Supplement: Supplementary file 1 [file plants-10-02297-s001.zip › plants-1400834-Supplementary.pdf]

Table S1. Nucleotide sequences of qRT-PCR primers.

Abbrev.	Forward	Reverse
BCAT1	CGCCTGCTGCAAATGGTAC	ACGCTCCTCAACCTTGAAACC
VPS	AGCCGGGCTGACATTTCACTT	GGCCTCAATCAAGCTCTTCTCAA
PT1L	TCCAGGCTCGAGAGTTGGAT	ACAACGTATTCCGCAGAGAAGAG
PT2	TCTTTGCATCTGCCTTAATCTTCA	GCTCTCACTGGCCTCCTTA
HS1	CGCCTGGCATCCAATGAC	GCGCAAGCTTTCTACCCAA
HS2	GGCATCCAATGACTCCCAATTTAG	TGCGTGTGCAAGCTTTCTAC
PAL	CAGGCCATCGACTTGAGACAT	GCTGGTCGTCAGCACTTTCTTAG
4CL2	TGTGGCACCGTAGTTAGGAA	CTCACCGGGCTCGTTATAGG
CHSH1	GGCTCACATTTCATCTCCTCAA	GGCCTCGACTAAGCTCTTC
OMT1	GCGGCTCCATATCAGAGCTTA	ATGCCGGTGCTGTGGCAAT
F3H	TCAACCGGACCTGACTCTA	GTCCTGTAGCAGAAGGGTTATGG
LAR	GAGGCGCATGGTTGAAGAG	GGCCAAGAAGCGATGGAGTT
GPPS-SSU	CACGCGGCCAACTTCACT	ACTTGGGTCGTAAGAATTGTGGATA
FPPS	CCACTTCACCATCGCATTGTTC	ACCAGCCATCACCAATGCACAAG
MTS1	ARTGCACTGGAGAATGMKAAGGTT	AGCAAGCTCGAGAAAAGTGG
MTS2	GAGATGGCTTGGCTTTCAATAGG	AGASTCTTTCGTTATGGGATTTGTG
STS1	TTCGCCTCACTTGCTGATGA	CCCACCTTTCAACTGCTTCA
STS2	ATTGCACTCACCTCAATTAC	CCTAGCACTCTGCTCAATCGC
TPS9	CATGGCAAGAACTGCCCAATTC	GTCGGTGCATGGTTTGACAATC
NES	CCAGCCGCTGAAGTGGTA	TGCGACAAGATTGGGTCTGT
MYB3	GCTCCAGTACGGGTCGGATA	CCTCGCTCCACCAATTCTCTA
MYB8	GGAGCAATAAACGTGGGAGATTG	AGGTGGCGTGGCTTATTGAC
MYB78	TACACGCCGGAGAACTCTA	GGCGAAACCTGAGTCCCAAA
bHLH2	GGGTCGGAGAAGAGGAAATTGAG	GGTGGCGATGCAGACTGAT
bHLH4	CTCACTCAGTTCAGTCATCCACTA	TGCTGCTGTTGAAGAACTTGATC
WRKY1	GGCCGTCAAGAACAACAAATTTCC	CCCTTCGTCTTTCGTTAACCTC
TFIIIA-F1	GAGGCCGCATGTATGTTCTG	
TFIIIA-F2	GGACCTCGATCACGCTACATTCAG	
TFIIIA-F3	CATATGCAAAGCCATTCTCTTGAG	
TFIIIA-R	GGCGAGTCAAGTGATCCTTTC	
RPL5	CCGTTGCTCAAACCGAATCA	AGGGTGCCTTTGGTACTACTC
TTG1	CTCTGGGAGGTTCGTGATAACTC	TCGCTGGTCTTGCTGTTGT
MYC2	GAAGAGGGAGTTAGCAAGTAAAGAC	CCCACCGTGGTTTGACATC
PIF4	CTGCGGGAGCTTTCAAGGA	CAGACCCACCAGAAGATGAAGTAAC
GAPDH	AAGCAAGGACTGGAGAGGTG	CCAGTGCTGCTGGGAATAAT
RH46	CCAACCTACTGGGCTTCGAC	CAGAATGGGTATGATCGGGC
